# Newcomer Women's Experiences with Perinatal Care During the Three-Month Health Insurance Waiting Period in Ontario, Canada

**DOI:** 10.1177/08445621221150620

**Published:** 2023-01-11

**Authors:** Jessica Pimienta, Sepali Guruge, Oona St-Amant, Cristina Catallo, Corinne Hart

**Affiliations:** 1 70379 Lawrence S. Bloomberg Faculty of Nursing, University of Toronto, Toronto, ON, Canada; 2 Daphne Cockwell School of Nursing, 7984 Toronto Metropolitan University, Toronto, ON, Canada

**Keywords:** Ontario, Canada, immigrants, intersectionality, maternal and child health, perinatal care, health insurance waiting period

## Abstract

**Background:**

The three-month health insurance waiting period in Ontario reinforces health inequities for newcomer women and their babies. Little is known about the systemic factors that shape newcomer women's experiences during the OHIP waiting period.

**Purpose:**

To examine the factors that shaped newcomer women's experiences with perinatal care during the three-month health insurance waiting period in Ontario, Canada.

**Methods:**

This qualitative study was informed by an intersectional framework, and guided by a critical ethnographic method. Individual interviews were conducted with four newcomer women and three perinatal healthcare professionals. Participant observations at recruitment and interview sites were integral to the study design.

**Results:**

The key systemic factors that shaped newcomer women's experiences with perinatal care included social identity, migration, and the healthcare system. Social identities related to gender, race, and socio-economic status intersected to form a social location, which converged with newcomer women's experiences of social isolation and exclusion. These experiences, in turn, intersected with Ontario's problematic perinatal health services. Together, these factors form systems of oppression for newcomer women in the perinatal period.

**Conclusions:**

Given the health inequities that can result from these systems of oppression, it is important to adopt an upstream approach that is informed by the Human Rights Code of Ontario to improve accessibility to and the experiences of perinatal care for newcomer women.

## Background & purpose

Perinatal care is vital for all pregnant women to help identify potential maternal and newborn risk factors, discuss management of pregnancy-related or pre-existing conditions with their healthcare professionals, and determine what steps to take to promote a healthy pregnancy (Barkensjö et al., 2018). In April 1994, the three-month waiting period for the Ontario Health Insurance Plan (OHIP) was implemented in response to Ontario's economic crisis and fears about medical tourism (Goel & Beder, 2012). With this policy change, newcomer women became ineligible for OHIP, even though they were expected to pay taxes from the time of arrival. At any given moment, estimates suggest that as many as 500,000 people in Ontario are without provincial health coverage due to their immigration status (Barnes, 2016). This has significant implications for pregnant newcomer women who require perinatal care.

OHIP has been administered by the Ontario Ministry of Health (MOH) under regulation 552 of Ontario's *Health Insurance Act*, since 1972 (King's Printer for Ontario, 2022a). To be eligible for OHIP, a person must be planning to make Ontario their primary place of residence based on their immigration statuses under the categories such as, Permanent Resident, Convention Refugee, Temporary Resident Permits, or work permits under the federal Live-in Caregiver program (King's Printer for Ontario, 2022b). The *Canada Health Act* provides coverage for all medically necessary services. The provinces and territories are then responsible for defining which services are considered medically necessary, thus warranting coverage under their health insurance plan. All newcomers to Ontario are subject to a three-month waiting period, with the exception of those covered under the Interim Federal Health Program (e.g., refugee claimants), which provides basic coverage until individuals become eligible for health coverage in their province or territory (Government of Canada, 2022b). In Ontario, services that are covered under OHIP include perinatal care; visits to the emergency room, walk-in clinics, and other healthcare professionals; diagnostic tests and surgeries; annual eye examinations for youth (aged 19 years and under) or older adults (65 years and over); and prescription drugs for individuals aged 25 years and under or 65 and older (King's Printer for Ontario, 2022b).

The literature shows that a lack of healthcare insurance adversely affects newcomer women seeking perinatal care. For example, between 2007–2010, medical records from two hospitals in Toronto showed that 80% of uninsured immigrant, refugee claimant, and undocumented or partially documented precarious status migrant pregnant women received inadequate prenatal care, and 6.5% received no prenatal care at all (Wilson-Mitchell & Rummens, 2013). Women without health insurance are more likely to experience unattended home births because of the associated costs with inpatient care (City of Toronto, 2013). These outcomes suggest an urgent health policy and practice issue in Ontario and in Canada.

Building on the first author's clinical practice experience as a maternal child nurse and the results of previous work, the objective of this critical ethnographic study was to examine the systemic factors that shape newcomer women's experiences with perinatal care during the three-month health insurance waiting period in Ontario, Canada. Systemic factors include economic, cultural, and political systems of power within society (Carspecken, 1996). These factors are important to explore as they intersect differently with various social identities, creating unique perinatal care experiences for newcomer women (Pimienta et al., 2022). In this study, we defined a “newcomer” as a person who has established residential ties with Canada, including protected persons, those who have applied for or received Refugee status, and those who have applied for or received Permanent Resident status within the previous 10 years.

## Methods and procedures

An intersectionality lens rooted in Black feminist scholarship (Collins, 1990, 2000; Crenshaw, 1989; 1991; Crenshaw et al., 1995) was selected to frame this study and help illuminate how social identities intersect with systemic structures like insurance coverage to shape the experiences of perinatal care for newcomer women awaiting OHIP eligibility. Intersectionality does not give primacy to one particular social identity over another, as focusing on a specific social identity has been identified as a reductionist approach to understanding the multiple dimensions that produce healthcare experiences and health inequities (Hankivsky & Christoffersen, 2008). Further, an intersectional framework refrains from essentializing those who have the same social identities (Hankivsky & Christoffersen, 2008). This reduces the risk that comes with assuming all individuals from a social group are the same (Morrow et al., 2008). An intersectional approach was useful for this study because of the diverse sets of identities the participants brought with them that influenced their experiences.

A critical ethnography method was used to help examine how societal structures are embedded in culture and become taken-for-granted norms, values and beliefs which, illuminate opportunities to promote social justice (Carspecken, 1996). Critical ethnographic research goes beyond the description of social life by highlighting systemic inequities and oppression, thereby creating opportunities for positive change, such as enhancing the wellbeing of groups experiencing these social inequities (Carspecken, 1996; Kincheloe et al., 2018). Further details related to the methods used in this study have been published elsewhere (Pimienta et al., 2022).

Semi-structured individual interviews were conducted from April to September 2019 with four newcomer women and three perinatal healthcare professionals in the Greater Toronto Area, Ontario, Canada. Newcomer women of 18+ years of age who speak, read, and understand English were included if they had arrived in Canada within the last 10 years, and had received perinatal care during pregnancy, labor, delivery, or after the delivery of their baby, while waiting for OHIP coverage. Healthcare professionals who provided perinatal care to newcomer women within the same time frame were also included. Interviews lasted 30–90 min. Participant observations were conducted at recruitment centers, participant interview sites, surrounding neighborhood areas, and during participant interviews. Conducting participant observations in places where newcomer women seek or receive perinatal care yielded valuable insight into the factors that shape newcomer women's perinatal care experiences. All interviews and observations were conducted by the first author, a maternal child nurse and master's of nursing student.

Purposeful and snowball sampling strategies were used to recruit participants (Sandelowski, 1995). Recruitment posters were placed in various agencies where newcomer women or healthcare professionals may be accessible, such as community health centers and midwifery clinics. Individuals who expressed interest in the study were provided a study information letter, and a phone call was set up to determine eligibility. Eligible and consenting individuals were interviewed in a private room either at a university or a local community center. The interviews were audio-recorded and transcribed verbatim. Field notes were taken during participant observation.

Participant recruitment and data collection began after approval by the Research Ethics Board at the first author's graduate institution (REB 2018-425). Informed consent was obtained after providing participants with information related to the study purpose, data collection processes, risks and benefits, confidentiality, and process for withdrawal from the study. If individuals decided to participate in the study, they signed the consent form prior to the interview. Data collection and analysis were conducted simultaneously, moving back and forth through Carspecken's (1996) five stage process with an intersectionality lens guiding the analysis. Carspecken's strategies for rigor were also employed in each stage of the project (Pimienta et al., 2022), such as a modified method of member checking where participants were asked to clarify and verify the meaning of what they had shared during each interview, and were invited to refine the verbal summary described by the interviewer at the end of the session. Peer debriefing was also employed by discussing results among the entire thesis committee. Data collection ended once the data provided sufficient saturation and richness for an authentic description of the participants’ experiences (Malterud et al., 2016).

## Results

**Figure 1.**
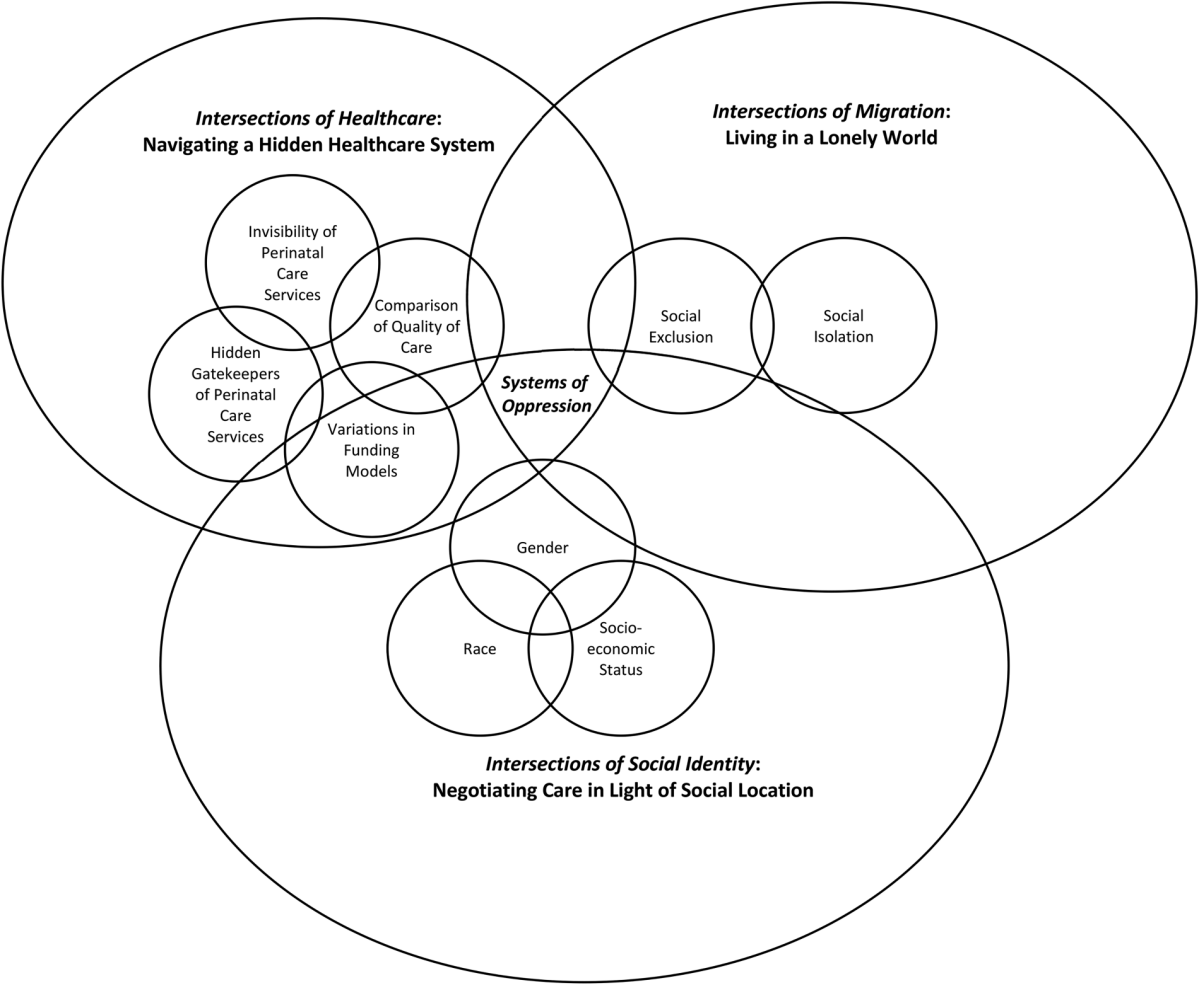
Systems of oppression: A framework for explicating perinatal health inequities.

In keeping with Carspecken's (1996) analytical approach, the results were based on conclusions drawn from analyzing both the observational and interview data. Carspecken (1996) suggests presenting the results first as “large categories” (p. 151) and then as more focused “intermediate categories” (p. 151), to unpack the broader issues. The overarching category derived from the data is: Systems of Oppression, which describes the three interconnected systems overlapping to create oppression for newcomer women. Three large categories were subsumed under the overarching category: 1) Intersections of social identity: Negotiating care in light of social location; (2) Intersections of migration: Living in a lonely world; and (3) Intersections of healthcare: Navigating a hidden healthcare system. The intersectionality diagram based on the work of McGibbon and McPherson (2011) was applied, and further developed in our study as a Framework for Explicating Perinatal Health Inequities (Figure 1). This diagram helps visualize how social identities and societal structures intersect synergistically to create unique forms of oppression for newcomer women in the OHIP waiting period. The diagram has three large circles of different sizes, representing the oscillation (back and forth movement) that happens when different identities occupy different degrees/levels of space in each individual's life. In the next sections, we have discussed the three main categories identified in this study (all participants have been given a pseudonym).

### Intersections of social identity: Negotiating care in light of social location

The largest circle represents the individual, where the forefront occupation of identity comes together to shape perinatal experiences. Emergent from the intersectional lens, various social identities, such as gender and race, intersect to create a social location, such as a racialized woman of low socio-economic status. Social location is comprised of both autonomous aspects of individual identity and interconnected systems of oppression. Social location combines with the immigration system and the healthcare system to create a unique form of oppression for each individual. The results from this study illuminate three aspects (intermediate categories) of social identity that create a cascading effect in an already oppressive healthcare system: gender, race, and socio-economic status. Each of these aspects occupies the forefront of identity at different times, shaping the perinatal care experience.

#### Gender

The results indicate that pregnant women are often viewed as a vessel with the primary purpose of carrying a baby. Many people feel that it is within their purview to weigh in on what is best for the baby, leaving women dissociated from their bodies. Gia, a newcomer woman, shared how they felt subtly pressured to simply follow the advice of a healthcare professional, despite wanting a referral to particular doctor.

*I had asked him [a healthcare professional] to be referred to this specific doctor, and he was like, “well, you can just go to hospital and just, you know, deliver”. If you don't understand the system, I could have just said “okay you’re the doctor, you know better. If this is what you think I need [for the baby], then so be it”*.

Lily, a midwife, discussed their experiences with other healthcare professionals who objectified women verbally in the presence of the client but also when clients were not present. This approach to care illuminates how perinatal care is situated within a patriarchal system, where discrimination against women renders them vulnerable. When this discrimination is paired with the idea of women as a vessel for a healthy baby and a lack of OHIP funded care, it renders pregnant women especially vulnerable.

*I see a lot of just mean-spirited treatment, which I think is really rooted in misogyny. Western bio medicines’ approach to women's bodies and to obstetrics, in general has been hostile, has been othering, like throughout history, and that's just woven into the fabric of Western biomedicine. When you’ve been educated in the western biomedical model, you see women's bodies as other, as disordered, as out of control. A lot of that latent underlying misogyny comes out in belittling language: Talking about my girl in room five, my gal who's a VBAC [Vaginal Birth After C-section], and that kind of language*.

The results also indicate that the objectification of women's bodies serves as a foundation for a default system of gender-based violence that is overlooked but sustained by various systems. For example, a patriarchal system reinforces a social hierarchy that produces inequalities in power for women, thereby maintaining gender oppression at the structural level. Blue, a midwife, discussed how gender-based violence may affect newcomer women, particularly, in the perinatal period by preventing access to perinatal care or services.

*Access to care for newcomer women is often episodic. Like if you’re living in fear of having to go back home to a powerful, dangerous husband, you can't make a refugee claim because then they would know where you [the newcomer woman] are. If they even access it [care] at all, they pop up when it's an emergency*.

Gender-based violence may also lead to ‘obstetric rape’, where healthcare professionals often perform medical exams and procedures without any regard for their client. Lily shared an experience with providing care for a newcomer woman who had been transferred to an obstetrician for a high-risk pregnancy.

*I mean, I’ve seen what gets called obstetric rape a lot in my career. Sometimes from nurses, mostly obstetricians. My client had a totally normal vaginal birth, but she experienced it as completely traumatic. No one introduced themselves; people didn't ask permission before they did internal exams. In obstetrics, it's so normalized to be able to touch people's bodies and to continue doing something when they’re saying “ow or stop”. Really, like making fun of the idea of consent*.

Lily's experiences of a patriarchal system were consistent with an observation the first author made at the end of a recruitment presentation she had at a community center.

*After the presentation, some individuals had questions about the study*.

*One woman raised her hand and asked if she could contact the first author at a later time, after getting permission from her husband to participate in the study.*

These results illuminate how gender-based oppressions at different levels create stereotyping and discrimination, for example, via objectifying language, and violence which can take on many forms, such as, physical, psychological, and economic.

#### Race

Participant interviews illuminated how racialized newcomer women are particularly vulnerable during the perinatal period. The healthcare professional participants noted that the majority of newcomer women they care for are racialized, that racialization has a significant effect on newcomer women during the perinatal period, and that racialized newcomer women often experience maternal health inequities as a result of systemic racism. For example, Lily said:

*This racism causes fatigue and a predisposition to chronic disease conditions. So with something like pregnancy, when there's stress on the system, stress on the body, the reason why like, we know that Black and Brown women have higher rates of hypertension, higher rates of diabetes, things like this*.

Blue, a midwife, also discussed how systemic racism, and the lack of OHIP funded care during pregnancy can lead to poor health outcomes. They highlighted how gender intersects with race to create systems of oppression for newcomer women.

*As soon as someone who has a Black body – it doesn't matter if they are a lawyer – when they’re naked in labour, a dehumanizing approach that is both misogynistic and racist comes into play*.

#### Socio-economic Status

In addition to gender and race, the data show that socio-economic status shapes the perinatal care experiences of newcomer women in the OHIP waiting period through the resources unavailable at the time of delivery. For example, Blue discussed how newcomer women who prefer to deliver in the hospital may not be able to choose this option due to the hefty deposit that is often required upon registration:

*Even within our midwifery world, the choice of birthing place is that one area that we see that finances thing come up over and over again. If you don't have the $500, the $1000, the $1500 a hospital is asking you to pay, you might choose a home birth*.

The inability to choose a hospital delivery – a privilege of OHIP coverage, results in a lack of access to valuable resources such as advanced resuscitation equipment, specialized care providers (such as, an obstetrician or pediatrician), or timely access to operating rooms (in the case that a cesarean section becomes necessary). Data also highlighted how socio-economic status can affect access to care throughout the perinatal period. Anja, a newcomer woman, discussed how she was not able to access free midwifery care and did not seek care by other healthcare professionals because she was unable to pay the deposit fees.

*So when I arrived here, because I did not have any OHIP coverage during the first three months, and I became aware that you have to make a deposit of like, $3,000 if I were to do it, I did not actually visit anybody*.

Healthcare professional participants suggested that food security and housing security are also affected by socio-economic status, which can have major implications for both the women and their babies. Newcomer women in the OHIP waiting period may have to pay fees for perinatal services (such as lab test fees, ultrasound fees, and hospital room fees), at the expense of essential items such as food and shelter. Blue explained:

*On the day of clinic, we’ll bring fresh fruit from the kitchen. And for some people, it's the first thing that they’ve eaten all day. It really dawned on us when we were getting ready to do a glucose challenge test for someone who hadn't actually eaten all day. It was an imperfect test*.

Access to adequate shelter can also have major health consequences for both mother and baby, particularly, in the postpartum period, where a safe and secure home is required to recover from the birth and care for the baby. Lily described that finding shelter spaces is a common struggle for newcomer women in the perinatal period who do not have access to financial and social resources:

*I have homeless newborns in my care. They’re Canadian newborns, they don't have housing. I had central intake tell me that a two-day postpartum parent and her baby could have a mat on a floor in an office from 7pm to 7am. And then would have to vacate during the day. That was all they had to offer*.

The data presented in this section provide examples where gender, race, and socio-economic status occupies the forefront of social identity. In these situations, social identity intersects with the immigration system and the healthcare system, to create oppression for newcomer women.

### Intersections of migration: Living in a lonely world

The second-largest circle represents the immigration system, whereby a newcomer identity occupies the forefront in shaping perinatal care experiences for newcomer women. The results from the study reflected two aspects (intermediate categories) that are affected by immigration status: social isolation and social exclusion.

#### Social Isolation

For many newcomer women, moving to Ontario means being separated from family members and friends who would have otherwise been there to provide support, particularly, in the perinatal period. Participant observations and interviews indicated that a lack of social support left newcomer women feeling isolated, which, in turn, affected their perinatal care experience. For example, the first author observed a participant walking into the interview room 20 min after the planned interview time.

*The participant apologized for being late and said: “I know I’m supposed to be on time, but the baby was up all-night crying so I could barely make it out the door. I don't have anyone to help me with the baby at night.”*

#### Social Exclusion

The results also show that immigration status creates a unique form of exclusion for newcomer women by precluding them from accessing perinatal care. For example, Liisa, a newcomer woman, discussed her experiences with trying to find a perinatal care provider during the OHIP waiting period. During this time, she walked into several clinics, and was turned down multiple times because of her immigration status.

*You know, I prefer to speak my home language. And I just try to go to Chinatown. Lot of advertising right, but when I keep calling, [and] some are saying, no, no more. No, no more new newcomers. But then one said, yeah, it's okay, but you need to pay*.

In some cases, newcomer women who are in the OHIP waiting period and have a high-risk pregnancy must pay thousands of dollars for perinatal care provided by an obstetrician. They may still be excluded from receiving care even if they are able to pay these fees. For example, Gia, a newcomer woman, recounted her experience of exclusion when trying to search for an obstetrician.

*When I called [potential obstetrician offices], they just wanted to make sure of how far I was in the pregnancy because he wasn't going to be seeing me if I was at the end of the third trimester. There were only two doctors that I could find who would take ‘cash patients’*.

Some healthcare institutions’ protocols related to OHIP coverage can also contribute to social exclusion, especially when these do not follow OHIP related legislation. For example, babies born in Ontario are entitled, by law, to receive an OHIP card. However, this is not always the case, as Lily recounted:

*The hospital had started refusing OHIP to babies whose parents, if one parent didn't have OHIP, which is against the law and not how OHIP was meant to be distributed to babies. But they had put it into the computer system so that it was automatic that a card wouldn't be issued if one of the parents didn't have OHIP.*

The healthcare institutions’ decision not to issue an OHIP card to babies born to parents awaiting OHIP status is a clear reflection of the default mechanisms in place that are geared towards exclusion of newcomer women and their babies.

### Intersections of healthcare: Navigating a hidden healthcare system

The smallest circle represents the oppressive healthcare system that all newcomers must navigate. Various aspects of the healthcare system intersect with individual identities to create unique experiences with perinatal care in the OHIP waiting period. These aspects (intermediate categories) include: invisibility of perinatal care services, hidden gatekeepers of perinatal care services, variations in funding models, and comparison of quality of care.

#### Invisibility of Perinatal Care Services

Newcomer women participants were often kept in the dark about what perinatal services were available, and how and where they could access them. As a result, the onus was placed on the participants to find these hidden perinatal services. Consequently, newcomer women in this study described how they navigated the healthcare system differently, with markedly different and inequitable outcomes in care. After a participant recruitment presentation, the first author took field notes on an informal conversation with a public health nurse who provides prenatal care classes at the community center.

*The nurse discussed how many women often walk into the centre unaware of the resources that are available to them and ask how they can receive healthcare during their pregnancy. It is unfair that many newcomer women are later in their pregnancy (usually after 20 weeks of gestation) when they are able to access healthcare, as compared to those who have OHIP status*.

#### Hidden Gatekeepers of Perinatal Care Services

In addition to the difficulties with finding available perinatal services, newcomer women in this study often faced individual actors (gatekeepers) in the system who present barriers to receiving care. Blue described how a lower billing rate was negotiated for clients who do not have health insurance and are not able to pay the set fees at some hospitals. However, not all clients were able to access these lower rates because of biased assumptions of wealth made by non-healthcare workers, such as office clerks.

*At the hospital, sometimes people will go in and say I would like the lower billing rate and then the clerk will, decide if they can get the lower rate based on say, if they [the newcomer woman] have an iPhone, or what kind of jewelry they’re wearing, or what kind of car they saw them pull up in*.

#### Variations in Funding Models

The healthcare professional participants highlighted that variations in healthcare funding models can also have a large impact on access to perinatal care for newcomer women*.* Lily described how a salary-based funding model in midwifery can help newcomer women access perinatal care.

*The [dominant] model of funding is still caseload volume driven. So here we became employees and we’re salaried. We can take people late to care. We can do partial care, so we can do like small bits of care for people, see them one time. We’re not tied to doing this whole course of care in order to get paid*.

This impact of salary-based funding is even more apparent in emergency situations, when the fees have not been negotiated prior to the client presenting to the hospital. Blue reflected on an experience where the lack of a funding contract endangered the life of their client and the client's baby.

*So we brought the client into hospital for what was meant to be a crash C-section, and it was delayed because the obstetrician at the time was in the hallway negotiating his fee with the client. It was one of those moments where like every minute counts, just get this baby out. Like it is not appropriate to be discussing the fees*.

#### Comparison of Quality of Care

In terms of the quality of care, both newcomer women and healthcare professionals reported that newcomer women who did have access to perinatal care, received high quality care. For example, Rose, a newcomer woman, said:

*They did a great job with it. So I brought a long piece of cloth back from home which is a very religious piece of clothing that you’re supposed to put on the baby as the baby is born, right, and so I told them to do it. And they were so perfect. Like, they did it as soon as the baby was born, they covered him in the blanket*.

In comparison to the perinatal care they had received in other countries, the majority of newcomer women participants preferred the care they received in Ontario. Gia talked about how emotional support from her healthcare providers heavily contributed to her positive delivery experience.

*I felt well taken care of, I felt people cared. I felt that people were trying their best to make me comfortable*.

Despite receiving high quality care, newcomer women felt that they had to comply with everything (e.g., allowing healthcare professionals in training to practice on them) so as not to disrupt or jeopardize their care. For example, Gia discussed how she was uncomfortable with a medical student performing a breast exam on her but did not voice this concern over fears of losing access to care.

*I remember them having like people in training and teaching them how to do a breast exam on me. It's like, I didn't feel comfortable being examined and I wasn't quite sure what to say, because you never know. Like, because you don't really know a lot about when can I say yes and when can I say no? I thought, I’m getting free care. I should just put up with it*.

Although healthcare professional participants recognized the high quality of care provided to newcomer women in Ontario, they also suggested that it requires improvement. Blue highlighted the lack of use of the “language lines” (phone lines that help with language translation) even when they are available, which is an added layer of exclusion that compounds the lack of OHIP coverage for newcomer women who do not speak English or speak ‘broken’ English.

*While the hospitals that I’ve worked at have all had access to a language line, very few of them will use them. And when you’re trying to impart really important information about their healthcare, like it's a real problem when you’re not using the language line, or like some form of interpretation*.

All of the above factors illuminate how Ontario's hidden ‘culture’ of lack of insurance coverage intersects with social identity and the immigration system to create forms of oppression for newcomer women awaiting OHIP status.

## Discussion

The results of our study highlight how social identities intersect with larger systems of oppression, creating unique challenges and vulnerabilities for newcomer women in need of perinatal care. More specifically, the results from this study show how Ontario's hidden healthcare system affects access to and the experiences of perinatal care, particularly, for racialized newcomer women who are of low socio-economic status, thereby exacerbating the challenges and vulnerabilities they experience by virtue of newcomer status. These results have particular implications for women awaiting OHIP coverage for health services in the perinatal period, as lack of access to timely healthcare services has been linked to adverse maternal and fetal health outcomes, such as, increased morbidity and mortality (Haddrill et al., 2014).

The idea of oppression as being related to health inequities is consistent with other literature that examined health inequities among Black gay and bisexual male youth in the United States (Harper et al., 2018), and Indigenous mothers in Canada (Herk et al., 2011). These studies highlight how oppression related to social identity can perpetuate health inequities for vulnerable groups across different contexts. This study expands the work of these and other researchers by illuminating how systems of oppression related to social identity, the immigration system, and the healthcare system can create health inequities for newcomer women without health insurance.

The notion of pregnant women as a ‘vessel,’ fulfilling a passive role in reproductive processes can be traced back to the biomedical model, grounded in a patriarchal healthcare system where male dominance is exerted over other social groups. In the current systemic context, healthcare professionals often make decisions about what pregnant women ‘need,’ leaving women with limited control over their perinatal experience (Cook & Loomis, 2012) by virtue of their gender. For racialized women, this experience is compounded by systemic racism, which refers to hidden biases that are present in systemic policies, practices, and processes that introduce and sustain racial inequity (Evans et al., 2020). The literature shows the long-term impacts of systemic racism to include poor perinatal outcomes, such as, maternal pre-eclampsia and neonatal hypoglycemia (Nguyen et al., 2012). These outcomes would clearly warrant critical healthcare services, which are normally covered by provinces under the *Canada Health Act* (King's Printer for Ontario, 2022b).

The results of this study suggest that discrimination against women within a patriarchal society creates and maintains a system of gender-based violence in the context of perinatal health care. This is consistent with other studies outside of Canada (Castro, 2019; Santiago, et al., 2018; Gray et al., 2019). Obstetric violence is a relatively new concept that emerged from activists in Latin America to describe both unintentional and intentional acts of mistreatment and abuse by perinatal care providers. Obstetric violence is a violation of human rights, and may include obstetric rape, such as performing episiotomies (a cut made in the perineum, typically to assist with difficult deliveries) without informed consent, excessive examination and touch of perineal areas, using forceful techniques for delivery without consent (e.g., performing suprapubic pressure for shoulder dystocia), and inadequate management of pain (Jardim & Modena, 2018). Accounts of women experiencing obstetric violence have been documented around the world, including Mexico (Santiago et al., 2018), Qatar (Gray et al., 2019), Brazil (Lansky et al., 2019), Kenya (Warren et al., 2017), Denmark (Schroll et al., 2013), and the United States (Diaz-Tello, 2016; Vedam et al., 2019). This study adds an additional dimension to notions of obstetric violence by showing how systemic structures create vulnerabilities and experiences of obstetric violence for newcomer women.

Participant observations and interviews indicated that many newcomer women belonged to a collectivist culture “back home”, which provides both physical and emotional support throughout the lifespan. Moving to Ontario, where the dominant culture is individualistic in nature, often results in social isolation and exclusion, which can influence levels of maternal stress and depression (Martinez-Schallmoser et al., 2005). Additionally, women of low socio-economic status who cannot afford to deliver in a hospital, and do not have equitable access to valuable resources and supports may be at increased risk for adverse perinatal outcomes, such as delivery of premature babies (Rosa et al., 2014).

Both newcomer women and healthcare professional participants recognized the culturally responsive care that is provided in Ontario. These results are inconsistent with literature that reports that many policies and procedures within healthcare institutions conflict with cultural beliefs and practices of newcomer women (Spitzer, 2004; Grewal et al., 2008; Reitmanova & Gustafson, 2008; Higginbottom et al., 2014; Lee et al., 2014), as the biomedical model dominates healthcare practice, leaving little room for ‘culture’ outside the dominant views and beliefs (Higginbottom et al., 2014). The discrepancy between our results and the literature may be attributed to the fact that participants in this study had either received care from a midwife, or provided care as a midwife. Midwifery care, in general, is situated within a profession that promotes safe and equitable health services (Association of Ontario Midwives, 2020).

The results also show that newcomer women are apprehensive about expressing concerns related to the perinatal care they receive for fear that it may jeopardize their access to care. This finding has both ethical and legal ramifications when considering informed consent, as individuals may consent to medical procedures or exams because of fear of losing access to care. Since newcomer women awaiting OHIP status already have reduced access to health resources, this fear essentially removes their sense of ability to advocate for themselves and their babies, question particular care outcomes or decisions made by healthcare providers, and/or make requests related to the care they receive, thus increasing their vulnerability.

Policies and protocols within healthcare institutions further exclude newcomer women from access to health resources. These results are consistent with other research that uncovered how babies born to parents who are not eligible for OHIP are denied registration for OHIP after birth, due to policies present in some Ontario hospitals (Bates & Cheff, 2018). Such policies are against the legislation in Ontario, which states that babies are entitled to OHIP coverage upon birth (King's Printer for Ontario, 2022c). Additionally, these policies are discriminatory because they place babies born in Ontario, who are legally Canadian citizens, at a disadvantage simply because of the identity of their parent/s. Delayed access to OHIP funded care can create health inequities for newborn Canadian citizens as parents may not be able to seek care until the infant has obtained OHIP status (such as in situations where parents cannot afford to pay for care). It can also create health inequities for new parents who experience high levels of stress as they navigate the system trying to obtain OHIP coverage for their children. Stress has been identified as a risk factor for many health issues, including coronary heart disease and/or stroke (Kivimäki & Steptoe, 2017), depression (Lee & Hsu, 2012), and high blood pressure (Sparrenberger et al., 2008).

Interestingly, the Ontario government created policies during the COVID-19 pandemic that shed light on the inequities that newcomers face as a result of immigration status. In an effort to curb the spread of COVID-19 in Ontario, the Ministry of Health made two policy changes related to OHIP coverage: temporary removal of the three-month waiting period (as of March 19th, 2020), and temporary funding for physician and hospital services for individuals who do not qualify for OHIP and do not have any other form of healthcare coverage (King's Printer for Ontario, 2020). As a result, any individual in Ontario could receive government-funded healthcare services during the pandemic. These policies will be removed at some point in the future (King's Printer for Ontario, 2020); therefore, this change could be used as a real opportunity to drive policy change to permanently remove the health insurance waiting period.

### Recommendations for change

The results of this study can inform nursing education, nursing practice, and health policy. In terms of nursing education, the study results reinforce the importance of adding content related to the Human Rights Code of Ontario (King's Printer for Ontario, 2021) to nursing curricula. This content can help clarify the issues of violence and equity related human rights violations when caring for newcomer women receiving perinatal care. Nursing educators, in collaboration with preceptors at partner healthcare institutions, can, for example, introduce reflexive exercises for students to complete before entering clinical settings to help explicate implicit biases and create a basis for open-minded, respectful, and equitable nursing care (Bennett et al., 2019).

In terms of nursing practice, nurses and other healthcare professionals must be encouraged to pay attention to and address unconscious stereotypes and prejudices that result in discrimination in order to combat health disparities among vulnerable individuals (Hostetter & Klein, 2018), such as newcomer women. Similar content related to the Human Rights Code of Ontario (King's Printer for Ontario, 2021) should also be incorporated into orientation programs for new nursing staff at practice settings. This might help new nurses identify situations where systemic discrimination exists within healthcare, and take action against these occurrences. To address the lack of social support for newcomer women in the OHIP waiting period, nurses could help set-up and facilitate in-person and/or virtual support groups for new mothers and families, in collaboration with the appropriate administrative staff.

In terms of health policy, the intersecting systems of oppression identified in this study would best be addressed by permanently removing the OHIP waiting period in Ontario. To target the systems of oppression related to social identities, such as gender, race, and socio-economic status, healthcare institutions should incorporate content related to the Human Rights Code of Ontario (King's Printer for Ontario, 2021) into policies that acknowledge the existence of biases related to social identity, even implicitly, and aim to reduce the impacts of discrimination related to these biases. For example, healthcare institutions can create policies that prioritize hiring of healthcare professionals from vulnerable groups and require all employes to attend anti-racism training and use inclusive language that promotes equity (e.g., not referring to clients as “gal”, as reported by healthcare professional participants in this study). In addition, the Canadian federal government should legislate all hospitals to adhere to a consistent set of lower billing rates for all individuals who do not have health insurance, to promote access to perinatal care for newcomer women of low socio-economic status. The systems of oppression related to Ontario's “hidden” healthcare system can be addressed by transitioning to a salary-based model of funding for midwives, which the healthcare professional participants highlighted as a better method to serve vulnerable populations, by providing flexibility to provide care at any point throughout the perinatal period and focus on securing community-based services/supports.

### Limitations and future research

The results of this study provide unique insights about the perinatal experiences of newcomer women awaiting OHIP status in Ontario. However, a limitation of the study is the exclusion of the experiences of newcomer women who do not speak English. Almost 600,000 people in Canada cannot hold a conversation in English or French, and there is likely a higher number who cannot navigate the healthcare system due to limited English language skills, and/or understanding of commonly used medical terminology (Higginbottom et al., 2015, 2016; Khanlou et al., 2017). Communication barriers can hamper early detection of pregnancy related/perinatal risk factors, contributing to situations of severe health complications that can lead to maternal morbidity and mortality (Urquia et al., 2015). Therefore, future research must include the perinatal care experiences among newcomer women who do not speak English or French and who are in the three-month OHIP waiting period.

Another limitation of this study is that all the healthcare professional participants were midwives, and the perspectives and experiences of other healthcare professionals might be quite different. Future research is needed to compare and contrast the views and the experiences of a range of healthcare professionals including obstetricians and/or family physicians providing perinatal care to newcomer women in Ontario and also other provinces. Such research would be useful in addressing issues of accessibility to perinatal care for newcomer women who are coping with a high-risk pregnancy and needing specialized surgical/gynecological training, as well as promoting best practices among perinatal care providers across Canada.

## Conclusion

Through the analysis of observational and interview data with newcomer women and perinatal healthcare professionals, this critical ethnographic study helped to illuminate three systems of oppression that intersect to shape perinatal care for newcomer women during the OHIP waiting period: social location, the immigration system, and the healthcare system. Intersectionality shaped our results by illuminating how these systems of oppression interact with one another, rendering newcomer women particularly vulnerable to a unique form of oppression that creates perinatal health inequities. The knowledge gained from this study can be used as a basis for implementing these recommendations in order to better support newcomer women that require perinatal care.
